# Therapeutic benefits of nanoparticles in stroke

**DOI:** 10.3389/fnins.2015.00182

**Published:** 2015-05-19

**Authors:** Stavros Panagiotou, Sikha Saha

**Affiliations:** Division of Cardiovascular and Diabetes Research, Leeds Institute of Cardiovascular and Metabolic Medicine, University of LeedsLeeds, UK

**Keywords:** blood-brain barrier, ischemic stroke, nanoparticles, drug delivery, diagnostic tool

## Abstract

Stroke represents one of the major causes of death and disability worldwide, for which no effective treatments are available. The thrombolytic drug alteplase (tissue plasminogen activator or tPA) is the only treatment for acute ischemic stroke but its use is limited by several factors including short therapeutic window, selective efficacy, and subsequent haemorrhagic complications. Numerous preclinical studies have reported very promising results using neuroprotective agents but they have failed at clinical trials because of either safety issues or lack of efficacy. The delivery of many potentially therapeutic neuroprotectants and diagnostic compounds to the brain is restricted by the blood-brain barrier (BBB). Nanoparticles (NPs), which can readily cross the BBB without compromising its integrity, have immense applications in the treatment of ischemic stroke. In this review, potential uses of NPs will be summarized for the treatment of ischemic stroke. Additionally, an overview of targeted NPs will be provided, which could be used in the diagnosis of stroke. Finally, the potential limitations of using NPs in medical applications will be mentioned. Since the use of NPs in stroke therapy is now emerging and is still in development, this review is far from comprehensive or conclusive. Instead, examples of NPs and their current use will be provided, as well as the potentials of NPs in an effort to meet the high demand of new therapies in stroke.

## Introduction

Stroke is a disease, which occurs unexpectedly and has a disastrous outcome. Approximately 15 million people will experience a stroke episode every year worldwide of which 33% is left with a permanent disability whereas ~40% of the cases will result in death (Go et al., 2014). Due to the high impact of the disease around the world, stroke is ranked as the second deadliest disease for individuals surpassing 60 years of age, and fifth among individuals of ages 15–59 (The European Stroke Initiave Executive Committee and the Eusi Writing Committee, 2003). The major issue concerning stroke is the lack of effectiveness of the current diagnostic tools. The coexisting etiological factors make it more difficult to determine the direct cause of the disease, and although the routine hematological and biochemical tests regarding stroke patients are carried out, hematological disorders cannot be accurately assessed. According to recent meetings of the American Heart Association, about 87% of strokes, appear to be cases of ischemic stroke (Go et al., 2014).

At the event of an ischemic stroke, the affected locus shows apoptosis of the cells. Despite the complexity of the events which occur during a stroke, the surrounding area (penumbra) can be rescued, as long as the appropriate drugs are administered in an early time frame (Zhang et al., 2015). In the molecular level, the oxidative stress caused by an ischemic event significantly affects the BBB. When the barrier's integrity is compromised the junctional complex formed by the endothelial cells allows for vascular fluid components (white cells, prostaglandins, amino acids etc.), to cross to the abluminal side of the brain. As these cells were never meant to exist within the central nervous system (CNS), it is believed that they contribute toward the progression of a vasogenic edema (Pillai et al., 2009).

The damage from an ischemic stroke does not apply only to the barrier's junctional complex, but also to the cells which form and support the blood-brain barrier itself. Lack of oxygen around the area of the endothelial cells (cell hypoxia) revealed up-regulation of the glucose transporter 1 (GLUT1) (Yeh et al., 2008). The endothelial cells are fortified by the presence of pericytes, which are important for the formation of new microvasculature, both during embryogenesis as well as in adult life (Bergers and Song, 2005). Additional support to the BBB comes from the astrocytes found in the brain. The astrocytic end-feet from these cells showed important Trans-Endothelial Electrical Resistance (TEER) increase when co-cultured with endothelial cells (Abbott et al., 2006). Chemical compromise of the astrocytes leads to a chain reaction where pericytes are immediately affected, occludin is reduced within the junctional complex, and the BBB is presented with increased permeability (Willis et al., 2004; Bonkowski et al., 2011).

## Treatments of today

The most important parameter during the event of a stroke is time. The activation of cells, which respond to brain injury or even cell death signaling, occurs in a matter of minutes. As previously mentioned the surrounding area which was affected by stroke can be salvaged and that is due to the indirect effect that the disease has on neighboring cells (Arai et al., 2011).

Drug administration to the area is desirable but less blood covering the area would also translate to less quantities of drug being delivered to the cells. Moreover, the BBB breakdown during a stroke is not an irreversible event. Mechanisms of cell repair are activated in order to restore the barrier's function (Liu et al., 2010) thus the direct administration of drugs in the brain is not ideal. At the moment there are developments in research in order to tackle the issue of drug delivery during stroke events, but the only commercially-available pharmaceutical agent is recombinant tissue plasminogen activator (rt-PA) (Jahan and Vinuela, 2009). The plasminogen activator allows the oxygenation of the damaged locus as well as partial restoration of the blood flow. This therapy is again highly depended on time in order to be effective (not as beneficial if it exceeds a 3–4 h window) but also increases the risk of bleeding if the damage is excessive (Messe et al., 2012). Provision of anticoagulants to the patients is the only other commercially available option but again anticoagulants are entirely used as precaution to future or suspected episodes, and in no circumstances can repair or shield the affected area from further damage (Chen et al., 2000).

## BBB breakdown after stroke and secondary neuronal damage

It has been previously shown that in an event of stroke, the BBB is severely compromised and incapable of re-establishing regular function for as long as 7 days post-trauma (Lakhan et al., 2013). The extended period of this breakdown could lead to further neuronal damage both from host as well as foreign components. During stroke a complex cascade of events is present (i.e., disruption of ion channels, excitotoxicity, inflammation, activation of apoptotic pathways). The occurrence of these events could lead to further neuronal damage (secondary neuronal damage) (Chen, 2012). The majority of this damage will not be detected in the early stages of a stroke; neuronal injury/cell death can progressively continue even months after the initial event (Dihne et al., 2002; Baron et al., 2014). Bacterial infection, head injury, stroke, or even autoimmune diseases can be involved in this secondary damage and lead to severe disorders such as motor impairments and neuropsychiatric illness (Chen et al., 2014). It has been previously demonstrated that the BBB may not be the only accessible barrier for effective brain injury treatment. According to Chen Y., monoisonitrosoacetone (which can cross the BBB with ease) does not reinstate the normal levels of aceltylcholinesterase (AChE) in the cholinergic nervous system. During an organophosphate (OP)-induced damage event, administration of therapeutic substances which are BBB-specific could lead to progression of a secondary neuronal damage and irreversible neurological deficits (Chen, 2012).

## Crossing the BBB during stroke

Due to the type of the disease, treatment needs to be target-specific where the administrated drugs will be able to preserve their molecular structure thus therapeutic abilities (bioavailability). The BBB has always been a constant boulder against efficient drug delivery. Usually “BBB-proof” drugs are characterized by increased lipophilicity and a small molecular structure/weight; regardless of the increased probability to successfully pass the BBB, studies have shown that drug delivery across the BBB has a crossing success rate of 4–7% (Sierra et al., 2011). In parallel with the issue of poor drug delivery, additional problems rise such as lack of systematic drug dosage, decomposition or even chemical absorption by the host's metabolic system (Ghosh et al., 2010). Increased rates of drug delivery can be achieved with vasodilation drugs. Stretching of the BBB junctional complex occurs which allows to molecules of higher molecular weight or different surface charge to cross to the abluminal side of the brain with minimal restrictions. Complete dissociation of the junctional complex also allows other molecules to penetrate the brain locus which are considered toxic to the brain and may cause more damage rather than protect during a cerebral edema (Sasaki et al., 1986; Beletsi et al., 1999). So according to the issues raised above, developing a drug for stroke disease must have high sustainability rates in order to go against the host's metabolism and natural defenses. Like in daily life, the transportation of goods by appropriately conditioned means, allows the product to stay in its initial form as well as in its optimum condition for a longer period of time. A similar delivery of goods into the brain can be achieved by the use of NPs.

## Drug delivery and NPs

A NP which is designed for drug delivery purposes can derive from materials with artificial characteristics or materials with an organic background. Micelles, inelastic spherical shells, nanotubular particles, liposomes, golden NPs, and polymers fall in the category of NPs (Mc Carthy et al., 2014). The use of the NP as an agent carrier can be achieved in various ways (encapsulation or conjugation with some of the host's components). Enhancement of the NP as a drug delivery element is accomplished by the determination of the NP's polarity, the introduction of a surface receptor recognized by endothelial cells/immunity cells (phagocytes), or the prolongation of their lipoid acid chain in an effort to increase lipophilicity (Mc Carthy et al., 2014). The thickness of the NP's capsule and the capsule's size are significant factors for increased therapeutic abilities. The sizes of NPs can range between 1 and 300 nm regardless of the type that is used. With variation in sizes the core to surface ratio changes. For example, smaller NPs have a smaller core to surface ratio, which allows a drug to be immediately released once the NP's membrane is breached. Larger NPs are not preferred due to the uneven drug release that may occur; inefficient drug delivery would take place if the NP is preventing its complete release due to either slow NP decomposition or entrapment of the drug within the particle's compartment (Singh and Lillard, 2009). The time of release for a drug is important, since an early release into the blood-stream results in decomposition by the host's metabolism and clearance from the host's system (Desai et al., 1997). Increased specificity regarding the destination of a drug can be achieved through the natural process of transcytosis. The versatility to adapt with other cellular components or be fused with them (i.e., antibodies, peptides) puts the NPs in a favorable position regarding their use as drug carriers.

## Examples of chemical agents that enhance NP efficiency

The surfacing of NPs with the polyethylene glycol (PEG) polymer creates a protective layer, which increases the lifespan of the NP in the circulatory system, and also enhances the NP's abilities to follow a transcytosis route (Xie et al., 2012; Alyautdin et al., 2014). NPs with a polysorbate coat have shown enhanced abilities to cross the BBB through endocytotic routes by binding to specific lipoproteins found on the surface of endothelial cells. The use of different polymers in combination, showed enhanced control regarding drug delivery and release. The use of polyethylene oxide (PEO) with lactic acid-*co*-glycolic acid (PGLA) showed that the desired drug can be found within the host's nervous system (Singh and Lillard, 2009). Targeting the transporters that are associated with the BBB is one of the best ways to deliver a drug in the CNS with high accuracy. For example, conjugation of liposomes with different molecular weights of PEG leads to the linkage of glucose with cholesterol; unavoidably the glucose-part of the fusion molecule is recognized by the brain endothelial cell transporter GLUT1 which grants access to the liposomes in a “Trojan horse” manner (Xie et al., 2012). The use of NP's is not limited to encapsulation of drugs or conjugation with surface receptors. Recent studies show that specific NP's can function as scavengers of reactive oxygen species (ROS). These free radicals increase during cerebral ischemia, especially after reperfusion. For example, platinum nanoparticles (nPts) appear to scavenge superoxide anions as well as hydrogen peroxide, when tested *in vivo* (Takamiya et al., 2012). The same scavenging properties are presented by ceria NPs; during cerium's oxidative changes (reduction—oxidization), oxygen binding occurs in a way that is similar to the biological antioxidants. These NPs can also be efficient in ultra-small scales (i.e., 4 nm) making them excellent candidates for treatments against stroke (Kim et al., 2012).

## NPs as a diagnostic tool in stroke

From the research that was recently performed, we can observe that future perspectives in regards to NPs and stroke therapy are promising. With the increasing number in types of NPs (Mc Carthy et al., 2014), parameters such as time of drug release, NP size, tolerance against degradation by blood components, will not be an obstacle toward drug delivery in an ischemic brain. Drugs that showed promising results *in vitro* such as statins or candesartan can now be encapsulated in NPs, which can use endocytotic or transcytotic routs in order to cross the BBB (Sierra et al., 2011; So et al., 2014). A combination of old and new ideas can be combined where labeled antibodies of CNS injury biomarkers such as S100 calcium binding protein B (S100B), vascular cell adhesion molecule (VCAM), glial fibrillary acidic protein (GFAP) etc., can be conjugated with NPs in order to quickly detect them using a computed tomography (CAT) scan or magnetic resonance imaging (MRI) (Jickling and Sharp, 2011). The ability of NPs to endure degradation could be used in order to circulate NPs, conjugated with blood clotting biomarkers in an effort of early diagnosis or even the disruption of a molecular cascade that leads to thrombosis (Jickling and Sharp, 2011). Steps toward the diagnostic use of NP's have been made by Kevin Y. Lin and his colleagues who have conjugated thrombin-sensitive peptide substrates on the surface of the NPs and can detect the changes of thrombin levels in the circulatory system providing this way constant monitoring for coagulation (Lin et al., 2013).

## Stroke therapy and NPs

Until very recently the NPs were used for experimental therapies, preclinical studies as well as early phase clinical trials (Dobrovolskaia et al., 2008; Jain et al., 2012). Despite of the current developments in NP research, the use of NPs in regards to CNS diseases (as well as stroke) is still under development. Studies show that cytidine 5′ diphosphocholine has neuroprotective abilities regarding reperfusion and ischemia (Hurtado et al., 2011). Encapsulation of this drug in the appropriate NPs could allow the drug to travel through the circulatory system into the CNS. Current studies show that encapsulation of the basic fibroblast growth factor (bFGF) or the caspase-3 peptide inhibitor (z-DEVD-FMK) into chitosan-NPs, showed high numbers of NPs crossing through the BBB(Yemisci et al., 2014). This was achieved by inducing a receptor-mediated transcytosis of the transferrin receptor-1, which is found on the endothelial cells forming the BBB. The great numbers of bFGF or z-DEVD-FMK found within the brain showed reduce blood loss after a 2-h artery occlusion in the middle cerebral locus. High success rates of the administration of the drug gave access to a 3-h therapeutic window (Yemisci et al., 2014). A more recent study by Alice Gaudin and associates showed that squalenoyl adenosine (SQAd) which is rapidly metabolized and cleared from the bloodstream, can preserve 75% of the initial administration intact in mouse plasma. Adenosine's hydroxyl groups are protected by the use of tert-Butyldimethylsilyl chloride (TBDMSCl) in order for the squalenoyl group to connect with the amino group of the adenosine. De-protection of the hydroxyl ends leads to nanoprecipitation and the formation of squalenoyl adenosine nanoassemblies (SQAd NAs). The ability of SQAd NAs to breach the CNS showed promising improvements of the neurologic deficit score in mice, which relates to cerebral ischemia (Gaudin et al., 2014).

## Summary

Although promising, the use of NPs in drug delivery is still under development and as previously mentioned, stroke initiates a series of events which can occur simultaneously or even for prolonged periods of time. While a compromised (open) BBB is not desirable, the 7 days post-trauma window where the BBB remains open can be used to cross NPs which could act as neuroprotective agents or even as diagnostic tools. From the studies that have been carried out, it was shown that NPs lose their efficiency as their size increases. Large NPs will either not be absorbed by the desired tissue, or the polymeric chain forming the NPs may end up blocking the recognition point of the NP from the desired surface receptor. Another obstacle is the undefined amount of drug that can be absorbed by a NP. If a drug is greatly absorbed, the amount of drug released or even the time of its release might not be beneficial in regards to drug delivery. As expected, inability of the NP to absorb the drug will result in the release of a significant amount which might not be beneficial if the desired drug is meant to be released as part of a chronic therapy (Singh and Lillard, 2009; Xie et al., 2012). Various alterations of the NPs could improve their efficacy in regards to drug delivery or their potential use in diagnostics/therapeutics (Table 1).

**Table 1:** **Modification of nanoparticles (NPs) to improve specificity against the central Nervous System (CNS)**.

**Modification of NPs**	**Target of modification**	**Use of NPs**
Introduction of a surface receptor	Adjusted polarity and lipopholicity	Drug delivery
Adjustment of size and thickness	Core to surface ratio	Drug delivery/release
Surfacing with PEG	Indurance and transcytotic pathways	Drug delivery
Liposome conjugation	Regognition by the GLUT1 transporter	Drug delivery
Vasolidation drugs (indirect)	Increased drug delivery in the CNS	Drug delivery
Compromised BBB (indirect)	Increased drug delivery in the CNS	Drug delivery
Specificity in the type (i.e., nPts)	Binding of ROS	Therapeutic
Conjugation with labeled antibodies	Detection through CAT/MRI	Diagnostic
Conjugation with clotting biomarkers	Monitoring thrombin levels	Diagnostic/Therapeutic

The latest developments in NP research show more types of polymers being used as NPs. One of the variables that can be altered in order to resolve the problem of low drug amounts crossing the BBB is the increased amount of NPs carrying the desirable drug. Increase of the dose will eventually result in increased toxicity toward the cells. M. Kolter et al., showed that significant reduction in cell viability won't occur unless dosages of 500 μg/ml or higher are used. Nevertheless, TEER is vastly affected with dosages as little as 15 μg/ml. By allowing the dissociation of the BBB we are also allowing the entrance of foreign (to the CNS) cells (i.e., white cells). This may lead toward a worsen outcome instead of an improvement (Kolter et al., 2015). Further adjustments of the parameters involved in NP research will eventually result in an optimized model, where NPs will be used as drug delivery vessels with minimum complications as well as high success rates.

To conclude, stroke is a time-sensitive disease and early diagnosis determines the patient's outcome. Unfortunately, stroke therapy is bounded by many limitations and its therapeutic window is significantly small. An effective compound should be able to provide neurological protection not just by targeting the BBB route but also through other CNS barriers in order to increase the potential of a neuroprotective agent in the brain. A spherical view of therapeutics should be adopted and take into account both primary and secondary neuronal damage. The advancements in NPs research can lead to their use as a diagnostic tool, a therapeutic tool or even the combination of both in order to prevent stroke from developing or leaving permanent neurological deficits.

### Conflict of interest statement

The authors declare that the research was conducted in the absence of any commercial or financial relationships that could be construed as a potential conflict of interest.

## Abbreviations

BBB: Blood-brain barrier
NPs: Nanoparticles
CNS: central nervous system
GLUT1: glucose transporter 1
TEER: Trans-endothelial electrical resistance
PEG: polyethylene glycol
ROS: reactive oxygen species.
